# P(VDF-TrFE) Polymer-Based Thin Films Deposited on Stainless Steel Substrates Treated Using Water Dissociation for Flexible Tactile Sensor Development

**DOI:** 10.3390/s131114777

**Published:** 2013-10-30

**Authors:** Hong-Jie Tseng, Wei-Cheng Tian, Wen-Jong Wu

**Affiliations:** 1 Department of Engineering Science and Ocean Engineering, National Taiwan University, Taipei 10617, Taiwan; E-Mail: hjtseng@mems.iam.ntu.edu.tw; 2 Department of Electrical Engineering, National Taiwan University, Taipei 10617, Taiwan; 3 Graduate Institute of Electronics Engineering, National Taiwan University, Taipei 10617, Taiwan; 4 Graduate Institute of Biomedical Electronics and Bioinformatics, National Taiwan University, Taipei 10617, Taiwan

**Keywords:** biomedicine, P(VDF-TrFE), tactile sensor, Traditional Chinese Medicine (TCM)

## Abstract

In this work, deionized (DI) water dissociation was used to treat and change the contact angle of the surface of stainless steel substrates followed by the spin coating of P(VDF-TrFE) material for the fabrication of tactile sensors. The contact angle of the stainless steel surface decreased 14° at −30 V treatment; thus, the adhesion strength between the P(VDF-TrFE) thin film and the stainless steel substrate increased by 90%. Although the adhesion strength was increased at negative voltage treatment, it is observed that the crystallinity value of the P(VDF-TrFE) thin film declined to 37% at −60 V. In addition, the remanent polarization value of the P(VDF-TrFE) thin film declined from 5.6 μC/cm^2^ to 4.61 μC/cm^2^ for treatment voltages between −5 V and −60 V. A maximum value of approximately 1000 KV/cm of the coercive field value was obtained with the treatment at −15 V. The d33 value was approximately −10.7 pC/N for the substrate treated at 0 V and reached a minimum of −5 pC/N for treatment at −60 V. By using the P(VDF-TrFE) thin-film as the sensing material for tactile sensors, human pulse measurements were obtained from areas including the carotid, brachial, ankle, radial artery, and apical regions. In addition, the tactile sensor is suitable for monitoring the *Cun*, *Guan*, and *Chi* acupoints located at the radial artery region in Traditional Chinese Medicine (TCM). Waveform measurements of the *Cun*, *Guan*, and *Chi* acupoints are crucial because, in TCM, the various waveforms provided information regarding the health conditions of organs.

## 1. Introduction

Recently, numerous research institutes have explored applications of ferroelectric materials, including lead zirconium titanate (PZT) [1,2], polyvinylidene difluoride (PVDF) [3–5], and polyvinylidene-trifluoroethylene (PVDF-TrFE) [6–8]. PVDF and P(VDF-TrFE) are polymer ferroelectric materials that offer the advantages of mechanical flexibility, biocompatibility, low crystallization temperatures, and a high piezoelectric constant. These materials are widely employed in sensing applications such as pressure sensors, tactile sensors, pyroelectric detectors, and thin-film transistors.

Various sensor types, including capacitive-based, piezoresistive-based, and piezoelectric-based sensors, are commonly used for tactile sensing. A flexible membrane and gap are typically included in capacitive-based sensors, which can be widely employed in mobile robot contact force arrays [9], pressure sensors [10,11], proximity sensors [12], and tactile sensing arrays [13]. For piezoresistive-based tactile sensors [14], applied pressure alters the resistance and can be employed for force sensors [15], pressure sensors [16], and tactile sensors [17]. Regarding piezoelectric-based tactile sensors, mechanical energy can be transformed into electrical energy by applying pressure. These sensors have the advantage of high sensitivity, improved hysteresis, excellent repeatability, and high durability and, therefore, are employed for human health biomedical monitoring. Other sensors, such as piezoelectric-based sensors [18], optical sensors [19], laser Doppler sensors [20], and CMOS image sensors [21], have been used to measure the physiological signals of the human body, such as the heartbeat, breathing, and pulse waveform at artery regions. Human body pulse waveforms can be monitored for diseases, such as cardiovascular disease and arteriosclerosis.

Conventionally, the surface treatment process for altering the surface contact angle involves plasma [22,23], laser [24], and heat treatments [25] to alter the contact angle of the stainless steel substrate surface. However, these treatment processes suffer from high costs, limited treatment areas, and high-temperature operations. In this study, a low cost DI water dissociation technique was used to treat the surface and alter the contact angle of stainless steel substrates at room temperature. In addition, the treated area can be large and only limited by the volume of the substrate container (currently an area of >100 cm^2^ stainless steel plate can be placed in the DI water dissociation system). This process increases the adhesion strength of P(VDF-TrFE) thin films on the stainless steel substrates.

After the DI water dissociation process, spin coating was performed to deposit a P(VDF-TrFE) thin film on the surface of flexible stainless steel substrates. A piezoelectric-based tactile sensor to monitor the rugged human body pulse waveforms at various artery regions was developed. It is intended to utilize the measurement results from commercial sensors to validate the waveforms measured from developed P(VDF-TrFE) tactile sensors in this work. The typical commercially available piezoelectric sensors are large in size and are difficult to measure multiple acupoints with small distance in 0.5–1 cm [26]. The developed flexible P(VDF-TrFE) tactile sensor can accommodate various topologies of the human body for arterial pulse measurements. In addition, the proposed sensors were fabricated through micromachining technologies and the individual sensor cell and the distant between sensor cells can be down to the micrometer scale. It is believed that these tactile sensors can be widely used for micro-electromechanical systems (MEMS) applications, such as energy harvesting [27], large area tactile sensors array [28], robot hands [29], pressure sensors [30], and fingerprint applications [31].

## 2. Experimental Design

### 2.1. Fabrication of a P(VDF-TrFE) Solution and Thin Film for Tactile Sensors

**Figure 1.**
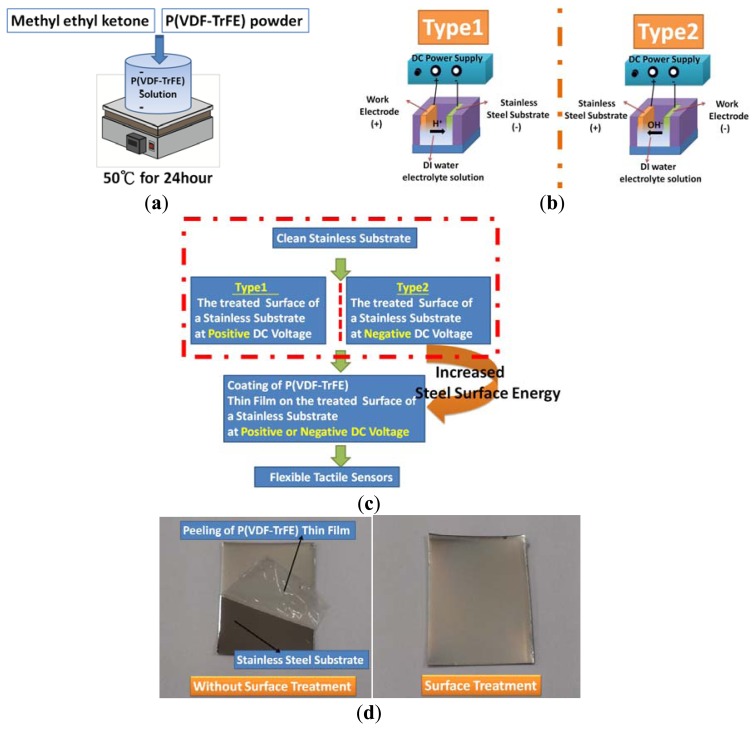
(**a**) Preparation of the P(VDF-TrFE) solution; (**b**) two types of DI water dissociation process for the surface treatment of stainless steel substrates; (**c**) the fabrication process of P(VDF-TrFE) thin film tactile sensors; (**d**) the P(VDF-TrFE) thin film can be easily peeled off from the underlying substrate without treatment (left) and adhered well to the underlying substrate with treatment by DI water dissociation system (right).

During spin-coating deposition, a P(VDF-TrFE) solution was spun onto flexible stainless steel substrates. As shown in Figure 1a, the P(VDF-TrFE) powder molar ratio of the two compounds obtained from PiezoTech SA (Saint-Louis, France) was 0.7:0.3. The powder was dissolved in a methyl ethyl ketone solution at 50 °C for 24 h. Then, as shown in Figure 1b, the substrate was cleaned ultrasonically in acetone, isopropyl alcohol, and DI water for 15 min. The substrate was then baked at 90 °C for 5 min before P(VDF-TrFE) thin film deposition. Subsequently, the stainless steel substrate was placed in the anode or cathode region. The DI water dissociation system comprised a DC power supply, a glass beaker, and DI water electrolytes. At an applied DC voltage of 0 to 60 V between the anode and the cathode, the hydroxyl group was attracted to the stainless steel substrate on the anode side, whereas, at an applied voltage of 0 to −60 V, the hydrogen group was attracted to the stainless steel substrate on the cathode side.

**Figure 2.**
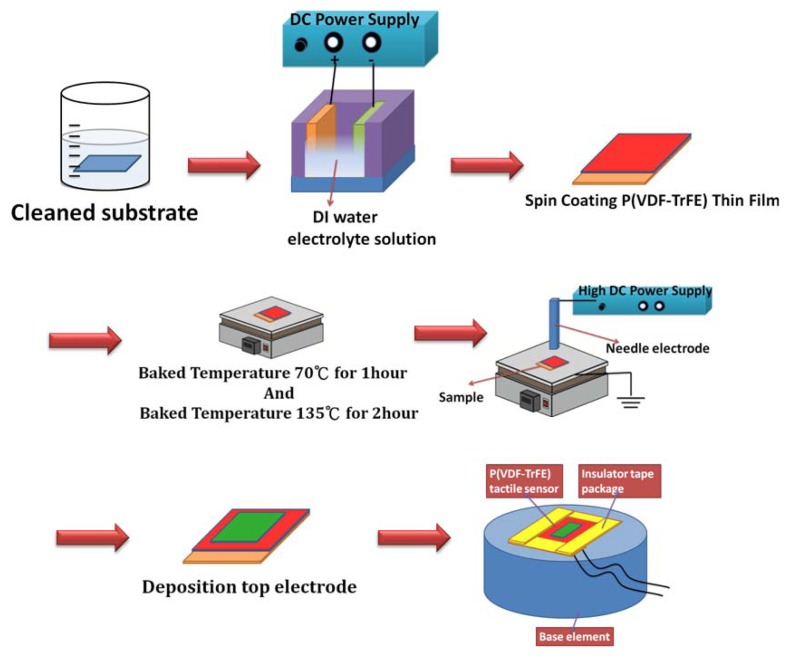
Fabrication processes of P(VDF-TrFE)-based tactile sensors.

Next, the P(VDF-TrFE) solution was spun onto the stainless steel substrate. The thin film then underwent baking at 70 °C for 1 h, followed by annealing at 135 °C for 2 h. The P(VDF-TrFE) thin film was exposed in the corona discharge process at 90 °C for 30 min and at room temperature for 15 min. Insulator tape was used to cover the sides and corners of the P(VDF-TrFE) thin-film tactile sensor and to secure the sensor to the base element. The fabrication and packaging processes are illustrated in Figure 2. Finally, the geometric specifications of P(VDF-TrFE)-based tactile sensors are shown in Table 1.

The DI water dissociation step is necessary for the fabrication of the tactile sensors. The fabrication process of the tactile sensors was shown in Figure 1c. By using the DI water dissociation process to treat the surface of the stainless steel substrate; its surface energy can be altered. As shown in Figure 1d, the P(VDF-TrFE) material has very low adhesion strength to the stainless steel substrate without surface treatment. With the surface treatment of the stainless steel substrate, the P(VDF-TrFE) material has great adhesion strength to the underlying substrate, as shown in Figure 1d.

Regarding surface treatment effects, the stainless steel substrates were examined using a contact angle meter (VCA Optima XE, AST Products, Inc., Billerica, MA, USA) to measure the contact angle of the substrate surface. The surface morphology of P(VDF-TrFE) thin films was examined using scanning electron microscopy (Hitachi, S4800, Tokyo, Japan). In addition, the surface morphology of the stainless steel was analyzed using an atomic force microscope (AFM, ThermoMicroscopes, ProScan, Sunnyvale, CA, USA). The crystal phase of the P(VDF-TrFE) thin film was analyzed using an X-ray diffraction system (BRUKER, D8-SSS, Berlin, Germany). The ferroelectric properties of P(VDF-TrFE) thin films were measured using a ferroelectric analyzer (Model TF2000, aixACCT, Aachen, Germany), and the piezoelectric coefficient value of P(VDF-TrFE) thin film was measured using a piezo d33 meter (PM3001, KCF Technologies, State College, PA, USA).

### 2.2. Tactile Sensor Experimental Design

**Figure 3.**
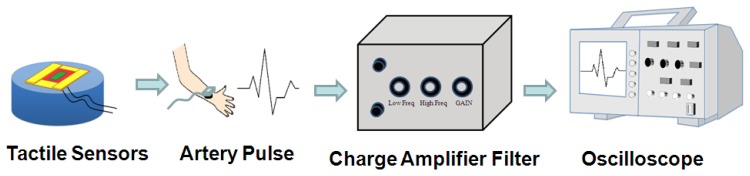
The Experimental set-up for P(VDF-TrFE) tactile sensor characterizations.

As shown in Figure 3, the P(VDF-TrFE)-based tactile sensors were used to measure the human pulse. Signals from the sensor were conditioned using a self-built testing system that comprised a charge amplifier (piezo film lab amplifier, Measurement Specialties, Hampton, VA, USA) and an oscilloscope (LeCroy 454 500-MHz WaveSurfer Oscilloscope, Agilent, Santa Clara, CA, USA). Various regions of the human body were selected for obtaining pulse measurements, including the carotid artery, brachial artery, finger, and ankle artery. A tactile sensor was connected to the charge amplifier to magnify the sensor signal, and a filter was used to eliminate undesirable signals outside the targeted frequency (1 to 10 Hz). Finally, the conditioned pulse signals were observed and recorded using an oscilloscope.

## 3. Results and Discussion

### 3.1. The Contact Angle of a Stainless Steel Substrate Surface Treated Using DI Water Dissociation, and Adhesion Tests of P(VDF-TrFE) Thin Film

**Figure 4.**
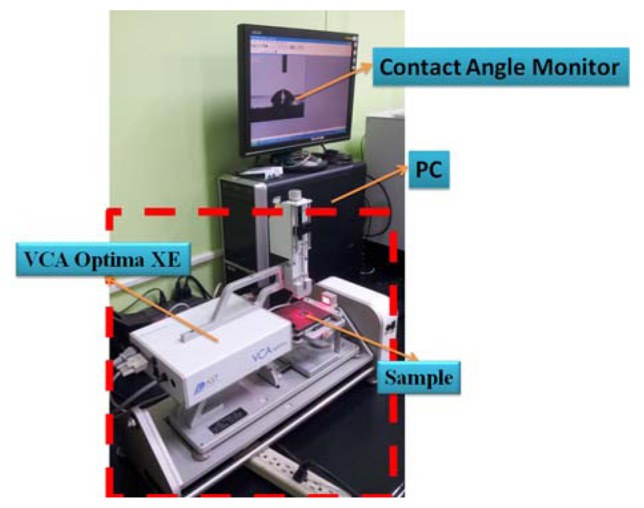
The experimental set up of the contact angle meter system.

**Figure 5.**
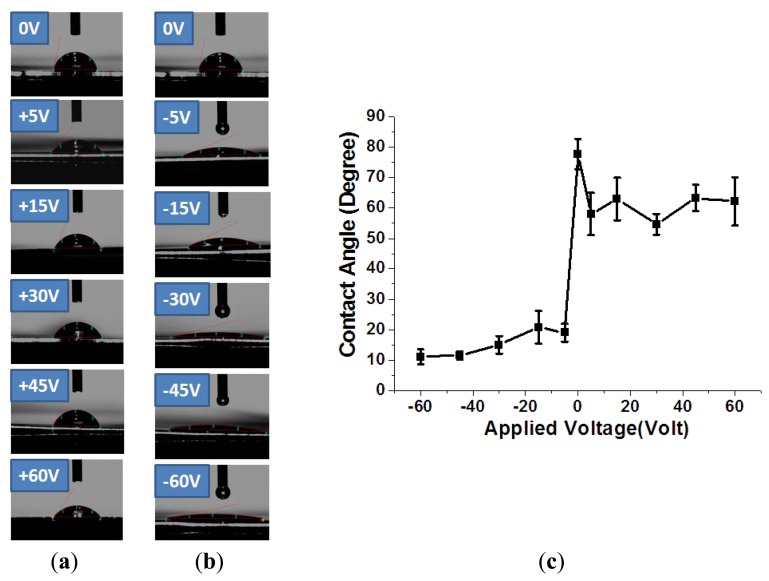
Contacle angles of the stainless steel substrate surface treated under different DC voltage settings of (**a**) 0 V ∼ +60 V and (**b**) 0 V ∼ −60 V with the proposed DI water dissociation process and (**c**) plots of the contact angles of substrate when applying anode (0 V ∼ +60 V) and cathode (0 ∼ −60 V).

A commercial system was utilized to measure the contact angle of the surface of the stainless steel substrate, as shown in Figure 4. This experimental information including the equipment information (VCA Optima XE, AST Products, Inc., Billerica, MA, USA), measurement conditions, the liquid type and the volume size of the drop, was shown in Table 2. As shown in Figure 5, the contact angle of the treated substrate surface was 77.8° at 0 V while the contact angle changed to approximately 60° with 60 V. When putting the stainless steel substrate in the cathode side, the contact angle of the treated substrate surface was 77.8° at 0 V, while the contact angle of the stainless steel substrate declined to 11.06° at −60 V. During the DI water dissociation process, the hydrogen and hydroxyl groups were respectively attracted to the anode and cathode regions by the DC voltage. The formation of the hydrogen and hydroxyl groups by the DC voltage during the DI water dissociation process can be expressed as follows [32]:

(1) $$2H_{2}O\to H_{3}O^{+}+OH^{-}$$

It is well known that a passivation layer of chromium oxide was formed on the surface of the stainless steel substrate. When 0 to 60 V of DC voltage was applied to the stainless steel substrate for 30 min, the hydroxyl group was attracted to the anode side. Simultaneously, these hydroxyl groups were oxidized to form oxygen on the anode side. This oxygen further reacted with the surface of the stainless steel to form a more uniform and complete chromium oxide layer. It is believed that this more uniform and complete chromium oxide enhanced the hydrophilicity of the surface of the stainless steel and thus the contact angle was reduced. When −60 to 0 V of DC voltage was applied to the stainless steel substrate for 30 min, the hydrogen group was attracted to the cathode side of the stainless steel substrate. These hydrogen groups will attack the chromium oxide on the surface of the stainless steel and an additional metal-hydroxyl layer will be formed. This additional metal-hydroxyl layer provided a stronger hydrophilicity and thus a larger decrease of the contact angle than the surface of the chromium oxide layer, as shown in Figure 5.

**Figure 6.**
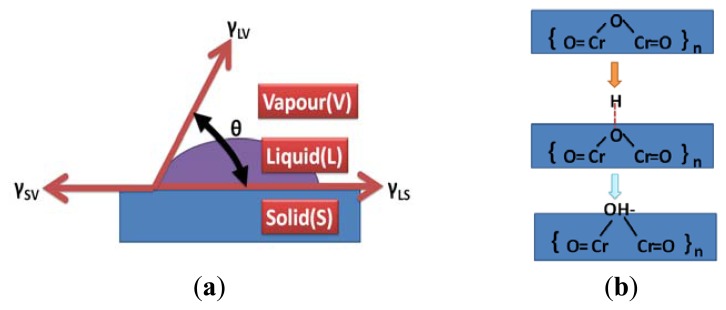
(**a**) Contact angle components on the substrate surface; (**b**) the hydrogen group was attracted to the cathode side of the stainless steel substrate under different negative DC voltage.

The total surface energy of the stainless steel substrate can be calculated based on Figure 6. The total surface energy can be calculated using the following formula [33]:

(2) $$\gamma_{\text{LV}}\text{cos}\theta =\gamma_{\text{SV}}-\gamma_{\text{SL}}$$

(3) $$\text{Wa}=\gamma_{\text{LV}}+\gamma_{\text{SV}}-\gamma_{\text{SL}}$$

Combining Equations (2) and (3) produces the following formula:

(4) $$\text{Wa}=\gamma_{\text{LV}}\left(\cos\theta +1\right)$$

where γ_SV_, γ_LV_, γ_SL_, and Wa represent the solid-vapor, liquid-vapor, solid-liquid, and total surface energy component, respectively. In addition, the liquid-vapor phase change for the water solution was 0.07197 N/m at 25 °C. As shown in Table 3, Equation (4) was employed to calculate the total surface energy of the stainless steel substrate; the approximate value was 0.0195 N/m at 0 V. Next, the total surface energy required to produce the maximum value was approximated to be 0.1418 N/m at −60 V. Thus, it was verified that DI water dissociation increases the surface energy of stainless steel substrates. During the DI water dissociation process, the hydrogen groups were attracted to the cathode side of the stainless steel substrate under a negative DC voltage, as shown in Figure 6b. These hydrogen groups will attack the chromium oxide on the surface of the stainless steel and an additional metal-hydroxyl layer will be formed to affect surface energy of the stainless steel substrate and thus to increase the adhesion strength between the P(VDF-TrFE) thin film and the underlying substrate.

**Figure 7.**
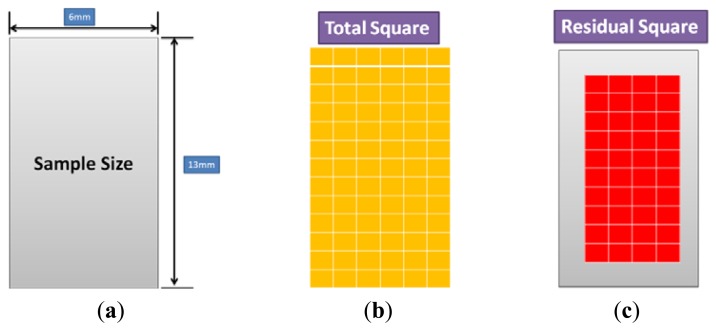
The adhesion testing results of (**a**) the P(VDF-TrFE) thin film on the stainless steel substrate before cutting; (**b**) array of squares was formed by cutting; (**c**) remaining squares after the detachment of the thin film using a 3 M scotch tape.

As shown in Figure 7a–c, the P(VDF-TrFE) thin film was cut into a square array on a stainless steel substrate with the length/width of approximately 13 mm/6 mm before the thin film adhesion testing. A knife was first used to scratch the film surface followed by the detachment of the thin film using a 3M Scotch™ tape. The calculation of the residual of the P(VDF-TrFE) thin film on the stainless steel substrate can be expressed as follows:

(5) Residual percentage(%)=Remaining squaresTotal squares×100%

**Figure 8.**
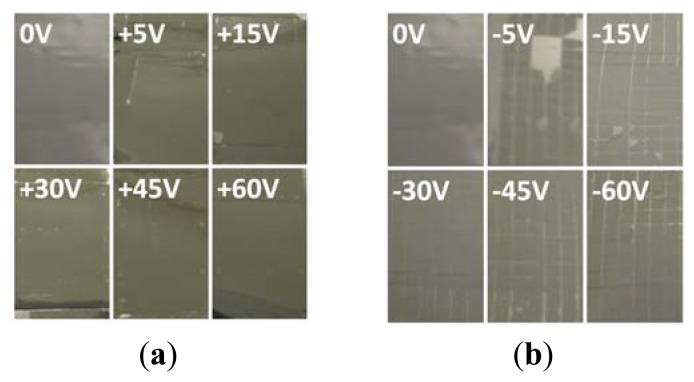
The adhesion strength test of the P(VDF-TrFE) thin film on treated stainless steel substrate surface under different DC voltage settings of (**a**) 0 ∼ +60 V and (**b**) 0 ∼ −60 V by the DI water dissociation process.

The P(VDF-TrFE) solution was spun on the treated surface of a stainless steel substrate. To characterize the adhesion strength of P(VDF-TrFE) thin film, the 3M Scotch™ tape was used to detach the thin film. A squares knife was first used to scratch the film surface. As shown in Figure 8, the P(VDF-TrFE) thin film adhesion test comprised two stages. During the first stage, DI dissociation surface treatment was performed on the anode side of the stainless steel substrate at 0 to 60 V for 30 min. When using the 3M Scotch™ tape to detach the thin film, the residual of the P(VDF-TrFE) thin film on the stainless steel substrate was approximately 0%, as shown in Figure 8a. When the DI dissociation surface treatment was applied to the cathode side of the stainless steel substrates at 0 to −60 V for 30 min, under the same adhesion strength test, the residual of the P(VDF-TrFE) thin film on the stainless steel substrate at −60 V was approximately 100%, as shown in Figure 8b. Thus, the surface treatment of stainless steel substrates using the DI dissociation system can increase the P(VDF-TrFE) thin-film adhesion strength and residual thin film by approximately 100%. The residual percentage of the P(VDF-TrFE) thin film on stainless steel substrate was between 0% and 2% without voltage or a positive DC voltage (0–60 V) while the residual percentage of the film was increased to 92% to 100% with a negative DC voltage (−5 to −60 V), as shown in Table 4. The increased adhesion strength is due to the change of the surface energy which was explained previously in this section.

### 3.2. The Crystal Orientation and Surface Morphology of P(VDF-TrFE) Thin Films

**Figure 9.**
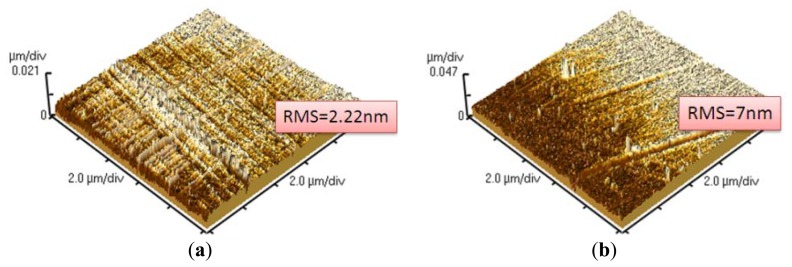
The surface morphology of the stainless steel substrate by (**a**) without and (**b**) with DI dissociation surface treatment.

**Figure 10.**
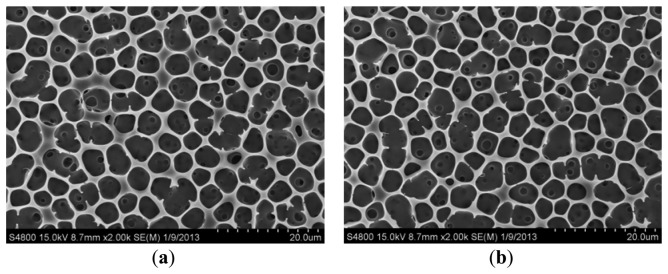
The P(VDF-TrFE) thin film deposited on (**a**) non-treated and (**b**) treated stainless steel substrate.

**Figure 11.**
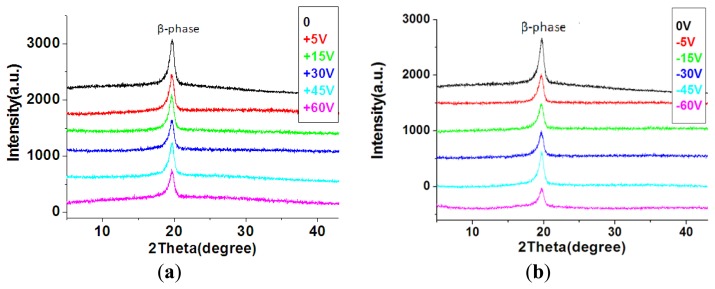
The crystal orientation of the P(VDF-TrFE) thin film on stainless steel substrates treated by various DC voltage values of (**a**) 0 ∼ +60 V and (**b**) 0 ∼ −60 V by DI water dissociation process.

The surface morphology of the stainless steel substrate treated with and without DI dissociation process was shown in Figure 9a,b. The root-mean-square (RMS) values of the measured roughness for the treated and non-treated surface were 2.22 and 7 nm, respectively. After the DI water dissociation process, the P(VDF-TrFE) thin film was deposited on the stainless steel substrate. There is no significant difference on the surface morphology of the P(VDF-TrFE) thin film deposited on either the treated or non-treated stainless steel substrate, as shown in Figure 10a,b. Porous structures on the thin films were formed due to the solvent evaporation from the P(VDF-TrFE) material [8,34]. The scan angle range of the X-ray diffraction system with filtered Cu Kα radiation (λ = 1.5406 Å) was 5° ∼ 45°. The major peaks of P(VDF-TrFE) thin film in the main directions of the β-phase peak occurred at 2θ = 19.6°, as shown in Figure 11.

**Figure 12.**
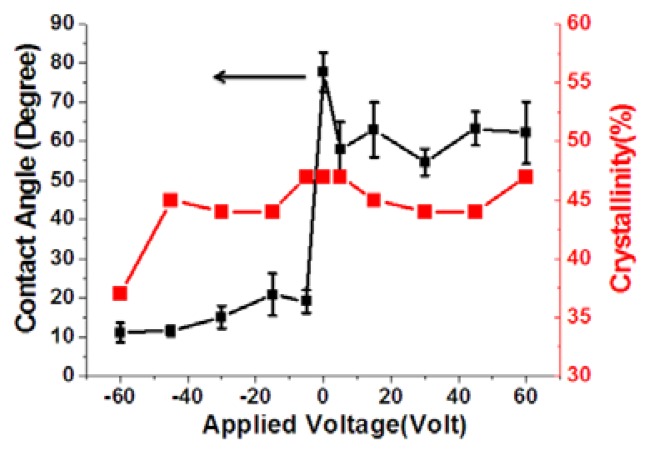
The relationship between the contact angle of the treated stainless steel surface at various voltage settings during the water dissociation process and the crystallinity of the P(VDF-TrFE) thin film.

**Figure 13.**
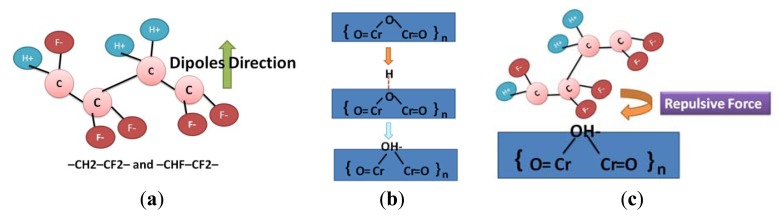
(**a**) The P(VDF-TrFE) material beta-phase structure; (**b**) formation of the metal-hydroxyl layer on the surface of the stainless steel substrate by DI water dissociation technique at the cathode side; and (**c**) a negative charged metal-hydroxyl layer and negative charged fluorine anions from the P(VDF-TrFE) material formed a repulsive force.

In addition, P(VDF-TrFE) thin film was deposited on the stainless steel substrate surface at various contact angles, which affected the crystallinity of the P(VDF-TrFE) thin film. The XRD patterns were obtained from the amorphous and crystalline peaks of the P(VDF-TrFE) thin film. The crystallinity equation can be expressed as follows [35]:

(6) $$\text{crystallinity}(\%)=\frac{A_{c}}{A_{c}+A_{a}}\times 100\%$$

where *A_a_* is the peak area of the crystalline phase, and *A_c_* is the peak area of the amorphous phase. As shown in Figure 11, the crystallinity of P(VDF-TrFE) thin films was not substantial (approximately 44% to 47%) at 0 to 60 V. In addition, the crystallinity values of the P(VDF-TrFE) thin film declined to 37% when the DC treatment voltages increased to −60 V, as shown in Figure 12. Because it is observed that the crystallinity of the P(VDF-TrFE) thin film, formed from P(VDF-TrFE) material as shown in Figure 13a [36], was affected by the surface energy of the underlying treated stainless steel substrate.

During the DI water dissociation process, the hydrogen groups were attracted to the cathode side of the stainless steel substrate, as shown in Figure 13b. These hydrogen groups will attack the chromium oxide on the surface of the stainless steel substrate and an additional metal-hydroxyl layer will be formed. It is believed that the negative charged metal-hydroxyl layer and negative charged fluorine anion from the P(VDF-TrFE) material formed a repulsive force and thus changed the crystallinity of the P(VDF-TrFE) thin film, as shown in Figure 13c. When using a positive DC voltage or a small negative DC voltage (< −45 V), the repulsive force is not strong enough to change the crystallinity of the P(VDF-TrFE) thin film. However, when a −60 V DC voltage was applied to the stainless steel substrate for 30 min, the increased density of the hydrogen groups and the thickened metal-hydroxyl layer provided a strong hydrophilicity on the stainless steel substrate which affected the crystallinity of the P(VDF-TrFE) thin film significantly.

### 3.3. Piezoelectric and Electrical Properties of P(VDF-TrFE) Thin Films

**Figure 14.**
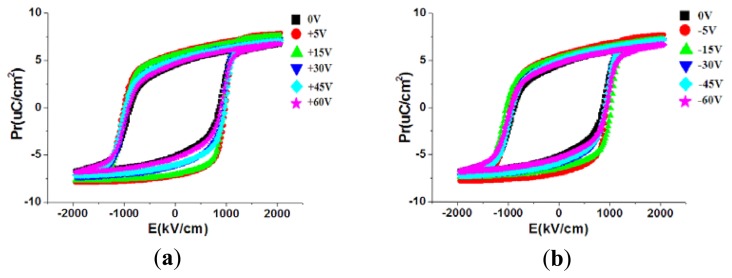
The D-E curves of the P(VDF-TrFE) thin film on the stainless steel substrate surface treated by the DC voltage values of (**a**) 0 ∼ +60 V and (**b**) 0 ∼ −60 V by the DI water dissociation process.

Figure 14 shows the measurement results of the hysteresis loops on the P(VDF-TrFE) thin film. The remanent polarization value of P(VDF-TrFE) thin films was 4.42 to 5.81 μC/cm^2^ at treatment voltages ranging from 0 to 60 V. The remanent polarization value of the P(VDF-TrFE) thin film declined from 5.6 to 4.61 μC/cm^2^ for treatment voltages between −5 and −60 V. The maximum remanent polarization value of 5.81 μC/cm^2^ was observed for treatment at 5 V. At a small applied voltage to measure DE curves, the curve trends were considered to be similar at either 0 to 60 V or 0 to −60 V. Therefore, the values of the remanent polarization at a small applied voltage were not deviated significantly (4.42–5.81) from each other. The main purpose to show Figure 14 is to prove that the P(VDF/TrFE) material was fabricated successfully by demonstrating the ferroelectric properties.

**Figure 15.**
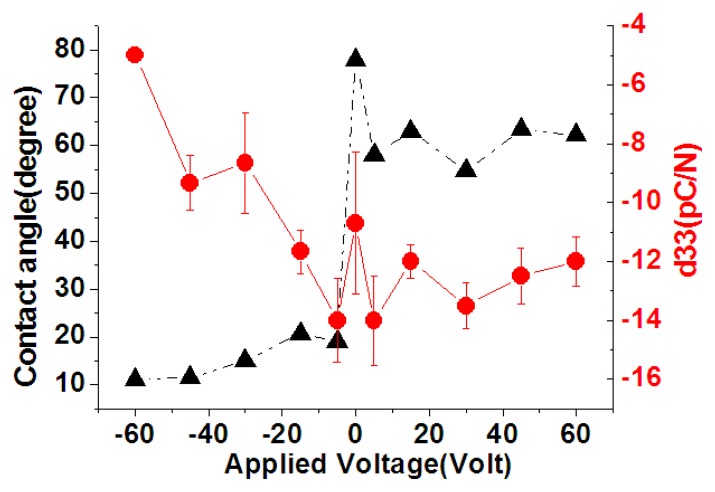
The relationship between the contact angle of the treated stainless steel surface at various voltage settings during the water dissociation process and the d33 of the P(VDF-TrFE) thin film.

As shown in Figure 15, the d33 coefficient value of P(VDF-TrFE) thin film was −10.7 to −14 pC/N for treatment voltages between 0 and 60 V. The maximum d33 coefficient value of −14 pC/N was found for treatment at 5 V. The d33 coefficient value of the P(VDF-TrFE) thin film decreased from −14 to −5 pC/N for treatment voltages between −5 and −60 V. Therefore, treating the stainless steel substrate surfaces by using the DI water dissociation method altered the surface water contact angles and thus the piezoelectric properties, was explained in Figure 13. The lower the contact angle of the stainless steel surface, the worse the crystallinity of the P(VDF-TrFE) thin-film formed above it. This decreased crystallinity of P(VDF-TrFE) thin-film lowered the d33 coefficient. The corona discharge poling system operated at 10 KV was utilized to measure the d33 coefficients. At a large applied voltage of >5 KV, the dipoles in the piezoelectric material will be aligned well to extract the representative d33 coefficients. From the measurement results, it is proved that a lowest d33 value was obtained at −60 V treatment compared with the d33 value at 0 V due to the difference of the crystallinity.

### 3.4. Pulse Measurements of Various Regions and Radial Artery Acupoints of the Human Body at Various Pulse-Taking Depths

#### 3.4.1. Monitoring Human Body Pulse Waves

For human pulse measurements, flexible substrates must be employed to accommodate the various surface topologies of the human body. Thus, flexible stainless steel substrates were selected because of their compatibility with the target piezoelectric material and greater robustness compared with other types of flexible materials (such as Al foil or polyimide). In addition, The DC treatment voltage applied on the stainless steel substrate to prepare the P(VDF-TrFE) film used in the tactile sensor monitoring human body pulses is −15 to −45 V. When the DC treatment voltage is between −15 to 60 V, it is observed that the P(VDF-TrFE) film will be detached from the stainless steel substrate during the human body pulse measurement. Therefore, it is very important to apply suitable treatment voltage to the stainless steel substrate for the tactile sensing applications.

The measurements conducted in this study (six arterial regions of the human body) were more diverse than the measurements conducted in previous studies because the sensor could detect most arterial regions of the human body regardless of its distance from the skin surface. Specifically, this tactile sensor was suitable for sensing pulse waveforms used in Traditional Chinese Medicine (TCM) monitoring. A comparison of the proposed sensor with other sensors is shown in Table 5.

**Figure 16.**
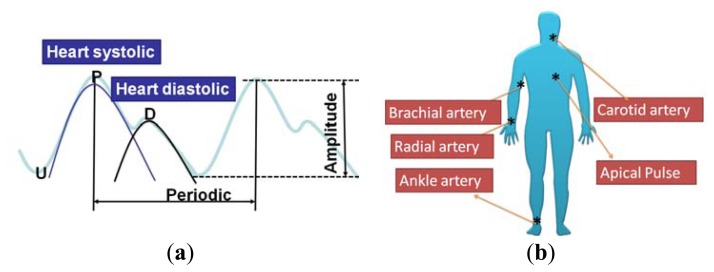
(**a**) The combination of the heart systolic and heart diastolic to produce the general human pulse waveform; (**b**) various artery regions for the tactile sensing in the human body.

As shown in Figure 16a, the average human body pulse waveform features two peaks; the first peak (in the P position) is the systolic blood pressure, and the second peak (in the D position) is the diastolic blood pressure. The period and magnitude of the human body pulse waveform are constant in a resting condition. These repeated signals were measured using the proposed tactile sensors at various regions of the human body, including the carotid artery, brachial artery, radial artery, ankle artery, and apical region, as shown in Figure 16b. The geometry and shape of the human body is typically irregular and complex. For example, the topology of the carotid region is smoother than that of the finger region, which enables easy pulse measurement. In addition, the amplitude of the pulse signal is highly dependent on the distance between the skin surface and underlying artery. Therefore, the flexible tactile sensor should be designed to be adaptable to a wide range of human body regions and to sensing pulses of varying amplitude.

**Figure 17.**
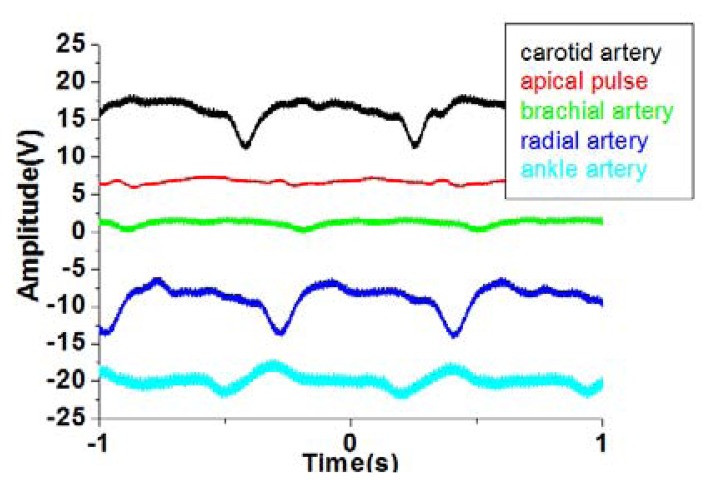
Comparison of pulse amplitudes and waveforms measured from numerous regions of the human body with our sensor.

As shown in Figure 17, P(VDF-TrFE)-based tactile sensors can sense pulses in various regions of the human body, from the carotid artery near the head to the ankle artery in the foot. Pulses from the carotid artery were measured first, and strong signals were obtained because of the smooth surface of the detected region. Next, the apical pulse from the heart was sensed to determine the heartbeat, and the waveform differed from that for the pulse monitored in other regions. In addition, weaker pulses were sensed in the apical pulse and brachial artery regions compared with the signals from the radial artery region. However, in the ankle region, the magnitude of the pulse is substantial because the ankle artery is extremely close to the surface of the skin. This study shows that the proposed P(VDF-TrFE)-based flexible tactile sensors can effectively detect various amplitudes and waveforms at different areas of the human body.

#### 3.4.2. Waveforms Corresponding to the *Cun*, *Guan*, and *Chi* Acupoints at Varying Pulse-Taking Depths

**Figure 18.**
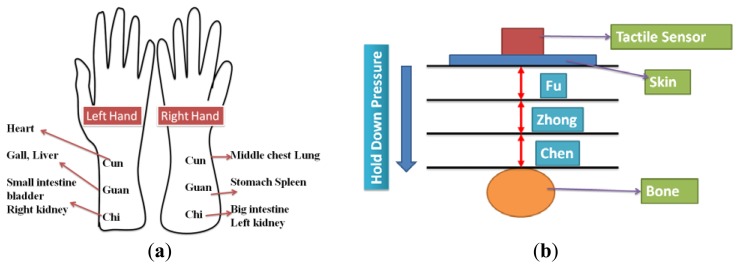
(**a**) A mapping of the *Cun*, *Guan*, and *Chi* acupoints to the corresponding organs in TCM; (**b**) the acupoints at different pulse talking depth of *Fu*, *Zhong*, and *Chen*.

**Figure 19.**
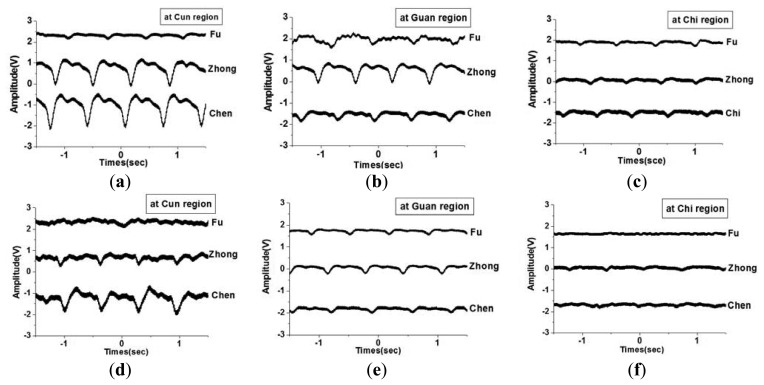
Waveforms of the P(VDF-TrFE) flexible tactile sensor to monitor the (**a**) *Cun*, (**b**) *Guan*, (**c**) *Chi* on the left hand, and (**d**) *Cun*, (**e**) *Guan*, (**f**) *Chi* on the right hand at different pulse taking depths.

The health condition of an organ can be determined based on the pulse waveform of the radial artery acupoints. In addition, depending on the pressure applied to the acupoints, the tactile sensors measure various pulses, and the waveform amplitudes and shapes differ accordingly. In TCM applications, three pulse-taking depths (three pressure amplitudes) applied to the acupoints are called *Fu*, *Zhong*, and *Chen*. The smallest pressure is applied when using *Fu*, whereas the greatest pressure is applied when using *Chen*. In TCM, monitoring the pulse waveform at the radial artery region provides substantial information for disease diagnosis. The three acupoints *Cun*, *Guan*, and *Chi* are located in the radial artery region, as shown in Figure 18a. The *Cun*, *Guan* and *Chi* acupoints were monitored at the pulse-talking depths of *Fu*, *Zhong*, and *Chen*, as shown in Figure 18b. In addition, the P(VDF-TrFE) tactile sensor was employed to monitor acupoints at various pulse-taking depths. The sensing depths of *Fu*, *Zhong*, and *Chen* are shallow, medium, and deep, respectively. As shown in Figure 19a to 19f, using the flexible P(VDF-TrFE) thin-film tactile sensors developed in this study, the pulse amplitude was greater in the left hand compared to the right hand when measuring the pulse waveforms of the acupoints on the radial arterial region. This indicates that the amplitude of the pulse waveform was greater in the comparatively stronger hand. Additionally, the pulse amplitudes at the same acupoint varied depending on whether *Fu*, *Zhong*, or *Chen* was applied. It is demonstrated successfully that these tactile sensors can be utilized for TCM applications.

## 4. Conclusion

This study demonstrates the successful fabrication and functionality of P(VDF-TrFE) flexible tactile sensors. DI water dissociation was used to treat the surface of stainless steel substrates and alter the surface contact angle, thereby increasing the adhesion of the P(VDF-TrFE) thin film to the stainless steel substrate. The decreased contact angle of the stainless steel surface changed the crystallinity of the P(VDF-TrFE) thin-film formed above it, and resulted in the change of the d33 coefficient of the film. The P(VDF-TrFE) thin film was deposited on flexible stainless steel substrates to provide broad pulse detection capabilities for various regions of the human body, such as the carotid region, brachial region, finger, ankle artery, radial artery, and apical region. The developed flexible P(VDF-TrFE) tactile sensor can accommodate various topologies of the human body for arterial pulse measurements. The amplitude of the human pulse waveform was high for the carotid artery, but low for the finger region. By using the P(VDF-TrFE)-based flexible tactile sensor developed in this study, a wide range of pulse sensing applications can be realized and used to monitor human health at various regions of the human body. Specifically, the developed P(VDF-TrFE) tactile sensor is suitable for monitoring the radial artery and *Cun*, *Guan*, and *Chi* acupoints precisely, which are used in TCM. Finally, the sensing depths when applying *Fu*, *Zhong*, and *Chen* can be employed to sense various waveforms at a given acupoint to further provide pulse information for TCM.

## Figures and Tables

**Table 1.** Parameters of the P(VDF-TrFE) thin film tactile sensor design.

**Tactile Sensor**	**Dimension**
P(VDF-TrFE) thin film	1 μm∼2 μm
Length	1.5 cm
Width	0.5 cm
Steel substrate thickness	100 μm
Top electrode thickness	100 nm
Top electrode length	1 cm
Top electrode width	0.3 cm

**Table 2.** Experimental parameters of the contact angle meter.

	**Experiment Parameter**
Contact angle meter	VCA Optima XE (AST Products, Inc.)
Measurement condition	Static measurement
Droplet type	Water
Droplet volume size	0.5 μL

**Table 3.** The contact angle and the total surface energy of the stainless steel substrate surface at numerous DC voltages.

**DC Voltage (V)**	**Contact Angle (degree)**	**Total Surface Energy (N/m)**
0	77.8	0.087
+5	58	0.11
+15	63	0.105
+30	54.66	0.114
+45	63.33	0.104
+60	62.2	0.106
−5	19	0.14
−15	20.75	0.139
−30	15	0.142
−45	11.45	0.143
−60	11.06	0.143

**Table 4.** The contact angle and total surface energy of the stainless steel substrate surface at numerous DC voltages.

**DC Voltage (V)**	**Residual Percentage**
0	0%
+5	0%
+15	0%
+30	2%
+45	0%
+60	0%
−5	92%
−15	97%
−30	100%
−45	100%
−60	100%

**Table 5.** Comparisons of various tactile sensors with different applications.

	**Substrate**	**Application**
PVDF [37]	NA	Radial
PVDF [38]	NA	Brachial, Radial
PVDF[39]	NA	Traditional Chinese Medicine (TCM) monitoring
AlN [40]	Polyimide (8.5 μm)	Finger
AlN [41]	Aluminum foils (1 μm, 16 μm)	Femoral
PZT [42]	Stainless steel	Carotid, Brachial, Finger, Ankle, Radial, Apical, PWV
P(VDF-TrFE) (this work)	Stainless steel	Carotid, Brachial, Finger, Ankle, Radial, Apical, Traditional Chinese Medicine (TCM) monitoring
